# Aflatoxin Risks in Ethiopia′s Food Supply Chain: A Systematic Review of Evidence From Maize, Groundnut, Red Pepper, and Milk

**DOI:** 10.1155/tswj/6290634

**Published:** 2026-11-14

**Authors:** Biadge Kefale, Nesru Zeynu

**Affiliations:** ^1^ Food Science and Nutrition Research Holeta Agricultural Research Center Ethiopian Institute of Agricultural Research Holeta Ethiopia eiar.gov

**Keywords:** AFB_1_, aflatoxin, AFM_1_, Ethiopia, food safety, groundnut, maize, milk, mycotoxin, red pepper

## Abstract

Aflatoxin contamination is a critical food safety challenge in Ethiopia, where climatic conditions and inadequate postharvest practices favor aflatoxigenic *Aspergillus* growth. This review synthesized aflatoxin occurrence, contamination levels, detection methods, and food safety risks in major commodities, including maize, groundnut, red pepper, and milk. A systematic review was conducted following PRISMA guidelines using peer‐reviewed studies identified from major databases. Data on commodity type, aflatoxin levels, analytical methods, and regulatory compliance were extracted and compared with international standards. Aflatoxin contamination varied widely among commodities and regions. Maize contamination ranged from nondetectable levels to 381.6 *μ*g/kg AFB_1_, whereas groundnut showed the highest contamination, reaching 1990.86 *μ*g/kg AFB_1_ and 11,900 *μ*g/kg total aflatoxins. Red pepper and berbere products contained total aflatoxins up to 98.99 *μ*g/kg, and milk AFM_1_ levels ranged from 0.003 to 4.98 *μ*g/L, with several samples exceeding the European Union limit. ELISA was the most commonly used screening method, whereas HPLC, UPLC, and LC‐MS/MS were used for confirmation. The findings demonstrate that aflatoxin contamination remains a significant food safety and public health concern in Ethiopia. Integrated strategies, including improved postharvest management, resistant crop varieties, feed safety control, surveillance, and stronger regulatory systems, are essential to reduce aflatoxin exposure.

## 1. Introduction

Aflatoxins are among the most important foodborne mycotoxins affecting global food safety, human health, and agricultural productivity. They are toxic secondary metabolites mainly produced by fungi belonging to the *Aspergillus* section *Flavi*, particularly *Aspergillus flavus* and *Aspergillus parasiticus*. These fungi can infect crops during preharvest development and continue producing toxins during drying, storage, processing, and distribution under favorable environmental conditions. Among the known aflatoxins, aflatoxin B_1_ (AFB_1_) is considered the most potent and toxic naturally occurring carcinogen and has been classified as a Group 1 carcinogen by the International Agency for Research on Cancer (IARC). Chronic exposure to aflatoxins is associated with hepatocellular carcinoma, impaired growth and development in children, immune dysfunction, and reduced animal productivity [1, 2]. Aflatoxin contamination is strongly influenced by environmental and agricultural conditions that promote fungal growth and toxin biosynthesis. High temperature, drought stress, insect injury, poor harvesting practices, inadequate drying, and improper storage are recognized as major risk factors affecting contamination throughout the food supply chain. Climate change is expected to further increase aflatoxin risks in many tropical and subtropical regions by creating more favorable conditions for *Aspergillus* infection and toxin production [3, 4]. Developing countries are particularly vulnerable due to limited postharvest infrastructure, inadequate quality control systems, weak regulatory enforcement, and insufficient access to advanced analytical facilities [5, 6].

Ethiopia represents one of the countries where aflatoxin contamination has become an emerging food safety challenge. The country has diverse agroecological zones and a rapidly expanding agricultural sector; however, traditional production systems, limited storage technologies, inadequate drying facilities, and insufficient routine surveillance contribute to increased contamination risks. Recent evidence indicates that aflatoxin contamination affects several important commodities in Ethiopia, including maize (*Zea mays* L.), groundnut (*Arachis hypogaea* L.), red pepper (*Capsicum annuum* L.), and dairy products [7–9]. Maize is one of the most widely consumed staple crops in Ethiopia and is also an important component of livestock feed systems. Contamination of maize with AFB_1_ and other aflatoxins poses a dual risk by directly affecting consumers and indirectly contributing to aflatoxin exposure through animal products. Recent studies have demonstrated substantial variation in maize contamination across Ethiopian regions, ranging from nondetectable levels to concentrations exceeding international safety limits, reflecting differences in agroecological conditions, storage practices, and analytical approaches [7, 10]. Groundnut is another highly susceptible commodity because its pod development occurs underground, increasing exposure to soil‐borne *Aspergillus* species. Studies from Eastern Ethiopia have reported extremely high AFB_1_ contamination levels in groundnut, with contamination strongly associated with genotype susceptibility, drought stress, delayed harvesting, and poor postharvest management [8, 11, 12].

Red pepper and its processed product berbere are culturally and economically important commodities in Ethiopia and represent a potential dietary exposure pathway because of their frequent consumption. Recent investigations have reported widespread aflatoxin contamination in dried pepper and berbere products, with contamination increasing along the value chain due to inadequate drying, storage, transportation, and market handling practices [13–15]. These findings highlight the importance of considering food processing and marketing systems as critical control points for aflatoxin management. In dairy production systems, aflatoxin contamination occurs primarily through the carryover of dietary AFB_1_ from contaminated animal feeds into milk as aflatoxin M_1_ (AFM_1_). Although AFM_1_ is generally less toxic than AFB_1_, it is classified as a possible human carcinogen and remains a significant concern because milk and dairy products are widely consumed, particularly by children. Several studies in Ethiopia have detected AFM_1_ contamination in raw milk, pasteurized milk, and dairy products, with some samples exceeding the maximum limit of 0.05 *μ*g/L established by the European Union [9, 16–18]. Importantly, conventional processing methods such as pasteurization do not completely eliminate AFM_1_ because of its relative thermal stability.

International benchmarks, including EU and Codex standards, provide reference points for interpreting aflatoxin contamination, although limits vary by commodity, intended use, and jurisdiction [19–21]. This review focuses on maize, groundnut, red pepper, and milk because they represent major components of Ethiopia′s food system and distinct aflatoxin exposure pathways. Maize is a staple food and livestock feed, groundnut is susceptible to fungal contamination, red pepper is vulnerable during drying and storage, and milk may contain AFM_1_ transferred from contaminated animal feed. Together, these commodities enable assessment of contamination across cereal, oilseed, spice, and dairy supply chains, comparison with international benchmarks, and identification of targeted interventions to improve food safety and reduce dietary exposure in Ethiopia.

Despite increasing scientific attention, existing evidence on aflatoxin contamination in Ethiopia remains fragmented across commodities, geographical regions, production systems, and analytical methodologies. Differences in sampling strategies, detection techniques, reporting units, and study objectives limit direct comparison among studies and hinder comprehensive assessment of national aflatoxin risks. Furthermore, limited synthesis has been conducted to evaluate contamination patterns across multiple food chains, compare reported concentrations with international regulatory standards, and identify critical intervention points. Therefore, a comprehensive synthesis of available evidence is essential to understand the magnitude and distribution of aflatoxin contamination in Ethiopia. This review is aimed at (i) summarizing the occurrence and contamination levels of aflatoxins in major Ethiopian food commodities, including maize, groundnut, red pepper, and dairy products, (ii) comparing reported contamination levels with international regulatory limits, (iii) evaluating analytical methods used for aflatoxin detection, and (iv) identifying research, technological, and policy priorities for reducing aflatoxin exposure and improving food safety in Ethiopia.

## 2. Methods

### 2.1. Review Design and Reporting Framework

This review was conducted following the principles of systematic review methodology and was reported according to the Preferred Reporting Items for Systematic Reviews and Meta‐Analyses (PRISMA) guidelines. The review focused on published scientific studies investigating aflatoxin contamination in food commodities and animal feeds produced, processed, or marketed in Ethiopia. The methodology was designed to systematically identify relevant studies, extract comparable information, and synthesize available evidence regarding contamination levels, analytical methods, and associated food safety risks.

### 2.2. Literature Search Strategy

A comprehensive literature search was conducted using internationally recognized electronic databases, including PubMed, Scopus, Web of Science, Google Scholar, and ScienceDirect. The search strategy was developed using combinations of relevant keywords related to aflatoxins, food commodities, and Ethiopia. The main search terms included (“aflatoxin” OR “aflatoxin B1” OR “aflatoxin M1” OR “mycotoxin”) AND (“Ethiopia” OR “Ethiopian”) AND (“maize” OR “groundnut” OR “pepper” OR “milk” OR “food commodities”). Additional relevant studies were identified through screening the reference lists of selected articles to ensure that important publications were not missed.

### 2.3. Eligibility Criteria

Studies were included if they met the following criteria: (i) The study was conducted in Ethiopia, (ii) aflatoxin contamination levels were quantitatively reported, (iii) the study investigated food commodities, (iv) sufficient information was provided regarding sample size, study location, analytical method, and contamination level, and (v) the study was published in a peer‐reviewed scientific journal. Studies were excluded if they did not provide quantitative aflatoxin concentration data and were review articles, conference abstracts, unpublished reports, or lacked essential information such as sampling location or analytical methodology. Studies with insufficient methodological descriptions were also excluded to maintain the reliability of the synthesized evidence.

### 2.4. Data Extraction

Data were extracted systematically from eligible studies using a standardized data collection format. The extracted information included publication details, sample size, study location, investigated food commodity, type of aflatoxin analyzed (AFB_1_, AFB_2_, AFG_1_, AFG_2_, AFM_1_, or total aflatoxins), reported contamination range, analytical detection method, and major findings related to contamination risk and food safety implications. Extracted data were organized into comparative tables to facilitate evaluation of contamination patterns among commodities and regions.

### 2.5. PRISMA Flow Diagram for Aflatoxin Studies in Ethiopia

Figure 1 presents a PRISMA flow diagram detailing the systematic selection process for studies on aflatoxin risks in Ethiopia′s food supply chain. From an initial 74 identified records, 16 duplicates were removed, and 33 were excluded during screening for reasons such as irrelevance to Ethiopia or aflatoxin, or being review articles. Following a full‐text assessment of 25 articles (excluding four more), a final total of 23 studies were included and categorized by commodity: maize (*n* = 4), groundnut (*n* = 5), red pepper (*n* = 5), and milk (*n* = 9).

**Figure 1:**
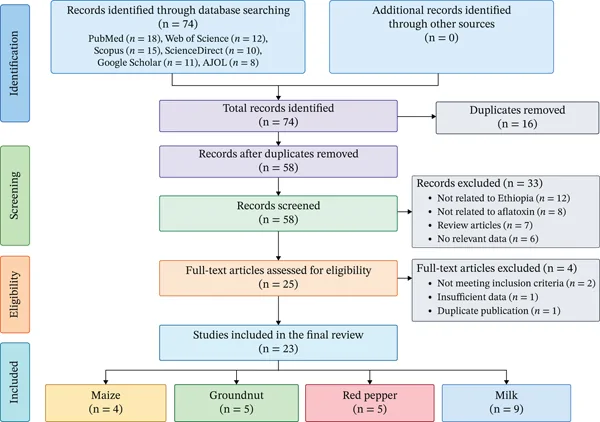
PRISMA flow diagram of study selection and distribution by commodity for aflatoxin risk in Ethiopia food supply chain.

### 2.6. Assessment of Aflatoxin Contamination Risk

The potential food safety risk associated with reported aflatoxin contamination was evaluated by comparing contamination levels from Ethiopian studies with internationally established maximum limits. The comparison included regulatory standards from the European Union, Codex Alimentarius Commission, and the US Food and Drug Administration. The proportion of reported contamination levels exceeding these limits was assessed to identify high‐risk commodities and critical points within the food supply chain requiring intervention.

### 2.7. Data Synthesis and Analysis

Due to considerable heterogeneity among studies in terms of investigated commodities, geographical coverage, sampling approaches, analytical methods, and reporting units, a narrative synthesis approach was applied. The findings were synthesized according to commodity type, contamination magnitude, geographical variation, analytical techniques, and food safety implications. Particular attention was given to identifying major factors contributing to contamination, including agricultural practices, postharvest handling, storage conditions, feed management, and limitations in monitoring systems. The review also highlighted opportunities for improved prevention, detection, and mitigation strategies to reduce aflatoxin exposure in Ethiopia.

## 3. Result and Discussion

### 3.1. Aflatoxin Contamination in Maize

Four studies investigated aflatoxin contamination in maize from different regions of Ethiopia (Table 1). Contamination levels varied considerably across studies. Yilma et al. [10] reported AFB_1_ concentrations ranging from not detected to 381.6 *μ*g/kg in maize samples collected from West Shoa and East Wollega, with 34.4% (31/90) of samples testing positive. In the West Gojjam Zone, Assaye et al. [22] reported total aflatoxin concentrations of 3.13–63.66 *μ*g/kg in preharvest maize and 9.02–139.80 *μ*g/kg in postharvest samples. Chauhan et al. [23] found total aflatoxin concentrations of 20–91 ppb using thin‐layer chromatography (TLC) and 28.24–91.40 ppb using a rapid immunochromatographic assay in maize from the Gedeo Zone. In contrast, Atnafu et al. [7] detected no AFB_1_, AFB_2_, AFG_1_, or AFG_2_ in 54 maize samples collected from Southwestern Ethiopia using liquid chromatography–tandem mass spectrometry (LC‐MS/MS). The studies employed different analytical methods, including ELISA, HPLC‐FLD, TLC, rapid immunochromatographic assays, and LC‐MS/MS. Overall, the findings indicate substantial variation in the occurrence and concentrations of aflatoxins in maize across the studied Ethiopian regions. The observed variation in maize aflatoxin contamination may reflect differences in agroecological conditions, fungal infection, harvesting practices, drying, and storage systems. The high maximum AFB_1_ concentration reported by Yilma et al. [10] highlights potential food safety concerns associated with contaminated maize, whereas the higher total aflatoxin range in postharvest samples reported by Assaye et al. [22] suggests that contamination may increase during postharvest handling and storage. However, these findings should be interpreted in the context of the studies′ sampling designs and analytical methods.

**Table 1:** Occurrence and detection of aflatoxins in maize grains from different regions of Ethiopia.

No	Sample size	Study location	Type of aflatoxin	Aflatoxin range	Detection method	Reference
1	150 maize samples	Gedeo Zone, Southern Nations, Nationalities, and Peoples′ Region (SNNPR), Ethiopia (Dilla Zuria, Yirgacheffe, Kochere, Qisha, and Wonago)	Total aflatoxins (AFB_1_, AFB_2_, AFG_1_, and AFG_2_ were targeted; AFB_1_ is the predominant toxin)	Immunochromatographic assay: Total aflatoxin 28.24–91.40 ppb (detection limit: 2–150 ppb); thin‐layer chromatography (TLC): Total aflatoxin 20–91 ppb (detection limit: 2–200 ppb)	Rapid immunochromatographic biosensor (Reveal Q+ Aflatoxin Test Kit with mReader) and thin‐layer chromatography (TLC)	[23]
2	54 maize grain samples	Southwestern Ethiopia (Jimma, Buno Bedele, and Ilu Ababora zones; nine districts)	AFB_1_, AFB_2_, AFG_1_, AFG_2_ (total aflatoxins)	Not detected (ND) in all samples	Liquid chromatography–tandem mass spectrometry (LC‐MS/MS)	[7]
3	30 maize samples (15 preharvest and 15 postharvest)	West Gojjam Zone, Amhara Region, Ethiopia (Burie, Finote Selam, and Jabitehnan woredas)	Total aflatoxins (AFB_1_, AFB_2_, AFG_1_, AFG_2_); AFB_1_ quantified separately	Total aflatoxins: Preharvest 3.13–63.66 *μ*g/kg; postharvest 9.02–139.80 *μ*g/kg. AFB_1_: Preharvest 3.13–27.31 *μ*g/kg; postharvest 2.83–29.50 *μ*g/kg	High‐performance liquid chromatography with fluorescence detection (HPLC‐FLD)	[22]
4	90 maize grains collected from six traditional storage systems	West Shoa Zone (Ilu Gelan and Bako Tibe districts) and East Wollega Zone (Gobu Sayo District), Oromia Region, Ethiopia	Aflatoxin B_1_ (AFB_1_)	0–381.6 *μ*g/kg (ppb); mean concentration = 0.2 *μ*g/kg; 34.4% (31/90) samples positive for AFB_1_	Enzyme‐linked immunosorbent assay (ELISA) using RIDASCREEN Aflatoxin B_1_ kit (Germany)	[10]

Chauhan et al. [23] demonstrated aflatoxin contamination in maize from the Gedeo Zone using both conventional TLC and a rapid immunochromatographic assay, supporting the potential value of rapid screening methods for routine monitoring. Conversely, the nondetection of aflatoxins by Atnafu et al. [7] indicates that contamination was not universal across the sampled locations. Nevertheless, nondetection does not establish the complete absence of aflatoxins, because results depend on analytical detection limits, sampling, and the representativeness of the samples. The reported concentrations also indicate potential noncompliance with applicable food safety standards. For accurate interpretation, results must be compared using the appropriate regulatory limit for the specific aflatoxin, commodity, and intended use. In particular, the European Union limit for AFB_1_ in maize intended for direct human consumption should not be confused with the limit for total aflatoxins. These findings emphasize the need for systematic surveillance, improved drying and storage practices, and effective aflatoxin control throughout Ethiopia′s maize supply chain.

### 3.2. Aflatoxin Contamination of Groundnut in Ethiopia

Groundnut is one of the major oilseed crops in Ethiopia with significant potential for domestic consumption and export markets. However, the reviewed studies demonstrate that aflatoxin contamination remains a critical food safety challenge, particularly in Eastern Ethiopia, where favorable environmental conditions such as high temperature, drought stress, insect damage, and poor postharvest handling promote infection by aflatoxigenic *Aspergillus* species. Across the five studies, AFB_1_ was the predominant and most frequently investigated aflatoxin, reflecting its high toxicity and classification as a Group 1 carcinogen by the IARC. The contamination levels varied widely depending on sample type, geographic location, storage conditions, and analytical approaches. Reported AFB_1_ concentrations ranged from 1.84 ppb in improved varieties [24] to 6.89 ppb in susceptible genotypes [11], indicating large genetic variation among groundnut materials (Table 2).

**Table 2:** Reported aflatoxin levels, study locations, and detection techniques for groundnut in Ethiopia.

No	Sample size	Study location	Type of aflatoxin	Aflatoxin range	Detection method	Reference
1	17 groundnut (*Arachis hypogaea* L.) genotypes (16 advanced breeding lines + 1 commercial cultivar, Werer‐961)	Babile Research Substation, East Hararghe Zone, Eastern Ethiopia	Aflatoxin B_1_ (AFB_1_)	13.98–1990.86 ppb (*μ*g/kg); 53% of genotypes had no detectable AFB_1_, whereas contaminated genotypes exceeded the African regulatory limit (10 ppb)	Enzyme‐linked immunosorbent assay (ELISA) using R‐Biopharm ELISA kit after methanol extraction and immunoaffinity column purification	[11]
2	28 groundnut (*Arachis hypogaea* L.) accessions/landraces collected from Ethiopian Biodiversity Institute (EBI), analyzed in triplicate, groundnut seed kernels (6‐month stored pods collected from traditional farming populations under rain‐fed conditions)	Oromia Region (Misrak Hararghe Zone: Babile and Gursum woredas; 20 accessions) and Benishangul‐Gumuz Region (Metekel and Kamashi zones; eight accessions)	Aflatoxin B_1_ (AFB_1_)	1.94–410.7 ppb (*μ*g/kg) AFB_1_. Lowest levels: 1.94 ppb (accessions 19,750 and 23,521); highest level: 410.7 ppb (accession 19,742). Only 6 of 28 accessions had ≤ 2 ppb, meeting the EU maximum limit	Indirect competitive ELISA using AFB_1_ ELISA kit (Product Code: KA07202H, Beijing Kwinbon Biotechnology Co. Ltd., China). Extraction involved acetonitrile–water/chloroform partitioning followed by ELISA quantification	[12]
3	145 *Aspergillus* section *Flavi* isolates obtained from groundnut kernels; 84 isolates tested for aflatoxin production	Eastern Ethiopia: Babile, Darolabu, Fedis, and Gursum districts (East Hararghe Zone); additional field samples from Babile	Aflatoxin B_1_ (AFB_1_), B_2_ (AFB_2_), G_1_ (AFG_1_), and G_2_ (AFG_2_)	AFB_1_: 0–77.98 *μ*g/mL (highest producer: *A. flavus* NPS N1436 from Gursum) AFB_2_: 0–0.26 *μ*g/mL (mean by isolates: 0.09–0.26 *μ*g/mL) AFG_1_: 0–50.33 *μ*g/mL (highest producer: *A. parasiticus* N1348 from Darolabu) AFG_2_: 0–0.13 *μ*g/mL	Ultraperformance liquid chromatography (UPLC) coupled with fluorescence detection (UPLC‐FLR) after chloroform extraction and purification using an aluminum oxide cleanup column	[25]
4	19 groundnut landraces/varieties (seven landraces from Gamo Zone, five varieties from Haramaya University, and seven varieties from Werer Agricultural Research Center); analyses performed using three replicates per sample. Ethiopian groundnut (*Arachis hypogaea* L.) landraces and released varieties	Gamo Zone (Boreda, Kucha, and Kucha Alpha woredas), Haramaya University (Haramaya), and Werer Agricultural Research Center (Werer)	Aflatoxin B_1_ (AFB_1_)	AFB_1_ (1.84–6.89 ppb)	Indirect competitive ELISA using AFB_1_ ELISA kit (Kwinbon Biotechnology, China) after extraction with acetonitrile/water and chloroform purification	[24]
5	120 groundnut samples (93 positive and 27 negative samples) collected from farmers′ stores and local markets	Eastern Ethiopia: Babile, Darolabu, and Gursum districts	Total aflatoxins (mainly including AFB_1_, AFB_2_, AFG_1_, and AFG_2_)	District‐specific ranges Babile: Store samples 157–11,900 *μ*g/kg; market samples 15–9770 *μ*g/kg Darolabu: Store samples 15–1980 *μ*g/kg; market samples 15–4940 *μ*g/kg Gursum: Store samples 15–5560 *μ*g/kg; market samples 16–10,100 *μ*g/kg	ELISA (enzyme‐linked immunosorbent assay) using RIDASCREEN Aflatoxin Total Enzyme Immunoassay kit (R‐Biopharm AG, Germany) after methanol extraction and immunoaffinity column cleanup	[8]

The highest contamination levels were reported by Chala et al. [8], where total aflatoxin concentrations reached 11,900 *μ*g/kg in samples collected from farmers′ stores and markets in Babile, Darolabu, and Gursum districts. These levels greatly exceeded international safety limits, including the European Union maximum level of 4 *μ*g/kg for total aflatoxins in food products and Codex guideline values. Such extreme contamination highlights the contribution of inadequate drying, delayed harvesting, mechanical damage, and improper storage conditions to aflatoxin accumulation. Recent studies indicate that contamination is associated not only with storage but also with genetic susceptibility of groundnut genotypes. Akale et al. [11] reported substantial differences among genotypes, with more than half showing no detectable AFB_1_, suggesting that resistant cultivars could play an important role in integrated aflatoxin management. Similarly, Syraji et al. [12] observed considerable variation among Ethiopian landraces, although most accessions exceeded recommended safety limits. These findings emphasize the importance of breeding programs targeting resistance to *Aspergillus* infection and aflatoxin biosynthesis.

The diversity of aflatoxin‐producing fungi also contributes to contamination complexity. Mohammed et al. [25] demonstrated that isolates belonging to *Aspergillus* section *Flavi* produced multiple aflatoxins, including AFB_1_, AFB_2_, AFG_1_, and AFG_2_. The presence of both *A. flavus* and *A. parasiticus* indicates that contamination management strategies should address fungal ecology throughout the production chain rather than focusing only on storage conditions. Analytical methods have evolved over time. Earlier Ethiopian studies mainly relied on ELISA‐based methods, which provide rapid and cost‐effective screening of large numbers of samples. More recent studies have incorporated advanced chromatographic techniques such as UPLC‐FLR, offering improved specificity and simultaneous detection of multiple aflatoxin types.

### 3.3. Aflatoxin Contamination of Red Pepper in Ethiopia

Red pepper (*Capsicum annuum* L.) and its processed product berbere represent important components of the Ethiopian diet and are economically significant commodities for domestic consumption and export markets. However, available studies demonstrate that aflatoxin contamination is a persistent food safety challenge throughout the pepper value chain, from field production to retail markets. The reviewed studies indicate high variability in aflatoxin contamination levels depending on production stage, geographical location, processing conditions, and analytical method. Mohammed [25] demonstrated that contamination increased progressively along the value chain, with relatively lower contamination during preharvest stages (ND–10.6 ppb) compared with farmer storage (0.3–17.1 ppb) and market‐level powdered pepper (3.1–19.2 ppb) (Table 3). This trend suggests that postharvest handling and storage conditions are major determinants of aflatoxin accumulation. The highest contamination levels were reported by Adugna et al. [13], where total aflatoxins reached 98.99 *μ*g/kg, with AFB_1_ concentrations up to 63.20 *μ*g/kg. Since AFB_1_ is classified as a Group 1 carcinogen by the IARC, these findings raise considerable concerns regarding chronic dietary exposure among Ethiopian consumers, particularly because berbere is consumed frequently and in relatively high quantities. Similarly, Satheesh et al. [15] reported that all analyzed berbere samples exceeded 20 ppb total aflatoxin, indicating widespread contamination across multiple value chain actors, including farmers, wholesalers, retailers, and processors.

**Table 3:** Occurrence, contamination levels, analytical methods, and geographical distribution of aflatoxins in red pepper products in Ethiopia.

No	Sample size	Study location	Type of aflatoxin	Aflatoxin range	Detection method	Reference
1	135 pepper samples collected from preharvest fields, farmer storage, and local markets (Burie, Finote Selam, and Jabitehnan districts)	West Gojjam Zone, Amhara Region, Ethiopia	Total aflatoxins (AFB_1_, AFB_2_, AFG_1_, and AFG_2_)	Preharvest: ND–10.6 ppb (mean 2.2 ppb); storage: 0.3–17.1 ppb (mean 10.7 ppb); local market powder: 3.1–19.2 ppb (mean 11.7 ppb)	ELISA using RIDASCREEN Aflatoxin Total kit (R‐Biopharm, Germany) after methanol–water extraction	[14]
2	18 dried red pepper samples collected randomly from markets (six districts); each sample ~1.5 kg	Amhara Region, Ethiopia: Central Gondar (Ayikel, West Dembia, East Dembia), South Gondar (Dera, Fogera), West Gojjam (Bure, Jabitehnan)	Aflatoxin B_1_ (AFB_1_), aflatoxin B_2_ (AFB_2_), aflatoxin G_1_ (AFG_1_), aflatoxin G_2_ (AFG_2_), and total aflatoxins	AFB_1_: 2.51–63.20 *μ*g/kg AFB_2_: 0.96–5.29 *μ*g/kg AFG_1_: 1.71–32.79 *μ*g/kg AFG_2_: 0.53–2.04 *μ*g/kg Total aflatoxin: 4.74–98.99 *μ*g/kg	High‐performance liquid chromatography (HPLC) equipped with a C18 reverse‐phase column and postcolumn photochemical derivatization. Sample extraction followed the AOAC method using methanol/water extraction and immunoaffinity column cleanup [26]	[13]
3	100 berbere samples (80 dried whole red pepper pods + 20 berbere powder samples) collected from different value chain stages (farmers, assemblers, wholesalers, retailers, industrial processors, and domestic processors)	Bure District, West Gojjam Zone, Amhara Regional State, Northern Ethiopia; additional value chain actors sampled from Bahir Dar City market	Total aflatoxin	All samples positive; total aflatoxin > 20 ppb (*μ*g/kg) (above the maximum recommended threshold)	ELISA method using VICAM AflaCheck test strips	[15]
4	Four sampling sites; 1 kg composite sample from Fiche market + 1 kg composite sample from Mukaturi market; additionally, 1 kg each of packed pepper powder brands (Afiya and Mudayi)	Fiche and Mukaturi towns, North Shewa Zone, Oromia Regional State, Ethiopia	AFB_1_, AFB_2_, AFG_1_, and AFG_2_ (total aflatoxins)	Raw red pepper: Fiche: AFB_1_ = 3.19, AFB_2_ = 0.19, AFG_1_ = 4.07, total AFs = 7.66–7.70 *μ*g/kg; Mukaturi: Not detected. Packed pepper powder: Afiya: AFB_1_ 7.01–7.06, AFB_2_ 2.11–2.20, AFG_1_ 0.50–0.51, total AFs = 10.4 *μ*g/kg; Mudayi: AFB_1_ 31.50–31.80, AFB_2_ 24.30–24.60, AFG_1_ 3.35–3.39, AFG_2_ 2.47–2.48, total AFs = 61.90 *μ*g/kg	Extraction with methanol:water (80:20, v/v); purification using immunoaffinity column (IAC); quantification by high‐performance liquid chromatography with fluorescence detection (HPLC‐FLD)	[27]
5	Three market samples of dried hot pepper (each sample 10 kg; subsampled into 12 experimental samples, 200 g each)	Markets in Ethiopia; irradiation and analysis conducted at Kality, Addis Ababa, Ethiopia, and Bless Agri Food Laboratory	Total aflatoxins (AFB_1_, AFB_2_, AFG_1_, and AFG_2_)	Control: 31.78–212.00 ppb; average initial aflatoxin: 97.48 ppb. After gamma irradiation: 2 kGy = 29.13–184.96 ppb, 4 kGy = 20.15–151.86 ppb, 6 kGy = 16.20–156.51 ppb	High‐performance liquid chromatography (HPLC) with fluorescence detection after immunoaffinity column cleanup and photochemical derivatization; AOAC official method 2005.08 used for extraction and cleanup	[28]

The occurrence of contamination at different supply chain points suggests that interventions should not focus only on farmers but should include storage facilities, transportation, processing environments, and market practices.

Commercially processed pepper products may represent an additional risk. Alemu and Geleta [27] found considerable differences between commercial brands, with one packed pepper powder product reaching 61.90 *μ*g/kg total aflatoxin, approximately three times higher than the maximum limit recommended by many international regulatory agencies. This finding highlights potential weaknesses in quality control systems, including raw material screening, drying practices, and storage management within processing industries. The effectiveness of postharvest mitigation technologies has also been investigated. Edae et al. [28] evaluated gamma irradiation and observed reductions in aflatoxin concentration; however, substantial residual contamination remained even after irradiation treatments. The results indicate that decontamination technologies alone may not completely eliminate aflatoxin risk, emphasizing the importance of preventive strategies such as improved drying, hermetic storage, biological control, and good agricultural practices.

### 3.4. Aflatoxin Contamination in Milk in Ethiopia

Aflatoxin contamination of milk and dairy products remains an important food safety concern in Ethiopia, mainly due to the carryover of AFB_1_ from contaminated animal feeds into milk as its hydroxylated metabolite AFM_1_. The reviewed studies demonstrate considerable variation in AFM_1_ occurrence and concentration among different geographical locations, dairy production systems, seasons, and analytical approaches. The earliest comprehensive investigation by Gizachew et al. [9] in the Greater Addis Ababa milk shed demonstrated substantial AFM_1_ contamination in raw milk. Among 110 milk samples collected from dairy farmers and milk collectors, AFM_1_ levels ranged from 0.028 to 4.98 *μ*g/L, with a median concentration of 0.094 *μ*g/L (Table 4). The same study identified extensive AFB_1_ contamination in dairy feeds, ranging from 7 to 419 *μ*g/kg, indicating that contaminated feed ingredients were the primary source of AFM_1_ contamination in milk. The strong association between feed contamination and milk contamination highlights the importance of controlling aflatoxin entry points at the animal feed level rather than focusing only on final dairy products.

**Table 4:** Distribution of aflatoxin contamination in milk across Ethiopia.

No	Sample size	Study location	Type of aflatoxin	Aflatoxin range	Detection method	Reference
1	100 raw milk samples from dairy farmers + 10 raw milk samples from milk collectors (total 110 milk samples)	Greater Addis Ababa milk shed, Ethiopia (Addis Ababa, Debre Zeit, Sebeta, Sendafa, Sululta)	Aflatoxin M_1_ (AFM_1_)	0.028–4.98 *μ*g/L (mg/L as reported in article); median 0.094 *μ*g/L	Commercial enzyme‐linked immunosorbent assay (ELISA) using Helica AFM_1_ kits	[9]
2	114 dairy feed samples from farmers + 22 feed manufacturer samples + 20 feed trader samples (total 156 feed samples)	Greater Addis Ababa milk shed, Ethiopia	Aflatoxin B_1_ (AFB_1_)	7–419 *μ*g/kg (mg/kg)	Commercial ELISA assay using Helica AFB_1_ kit	[9]
3	108 samples, milk (raw and pasteurized milk from local and industrial producers)	Bishoftu Town, Oromia Regional State, Ethiopia	Aflatoxin M_1_ (AFM_1_)	0.029–2.159 *μ*g/L (mean: 0.835 *μ*g/L)	ELISA (Helica Aflatoxin M_1_ Assay kit; competitive enzyme immunoassay)	[18]
4	114 cow milk samples (60 raw milk samples from dairy farms and 54 pasteurized milk samples from different brands)	Addis Ababa milk shed area, Central Ethiopia (Sebata and Sululta districts, Oromia Region)	Aflatoxin M_1_ (AFM_1_)	0–0.893 *μ*g/L (overall); mean = 0.0465 *μ*g/L. Raw milk: 0–0.893 *μ*g/L; pasteurized milk: 0–0.119 *μ*g/L Sebeta: 0–0.893 *μ*g/L; Sululta: 0–0.2018 *μ*g/L	High‐performance liquid chromatography (HPLC) using a C18 column with a fluorescence detector after immunoaffinity column cleanup	[17]
5	12 raw cow milk samples (500 mL each) collected from milk distributors in four kebeles (Bake Jama, Burka Jato, Cheleleki, and Bakanisa Kese)	Nekemte City, East Wollega Zone, Oromia Region, Ethiopia	Aflatoxin M_1_ (AFM_1_)	0.01–0.35 *μ*g/L (Bake Jama: 0.01–0.03 *μ*g/L; Burka Jato: 0.31–0.35 *μ*g/L; Cheleleki: 0.19–0.21 *μ*g/L; Bakanisa Kese: 0.03–0.07 *μ*g/L)	High‐performance liquid chromatography (HPLC‐FLD) after immunoaffinity column cleanup	[29]
6	45 milk samples	Holeta (Western Shoa): 15 milk Bishoftu (Eastern Shoa): 15 milk Hawassa (Southern Shoa): 15 milk	AFM_1_ (in milk)	Milk (AFM_1_): 0–0.146 *μ*g/L	ELISA (enzyme‐linked immunosorbent assay)	[30]
7	240 raw cow milk samples (120 samples collected during wet season and 120 during dry season)	Dale and Hulla districts, Sidama Zone, Southern Ethiopia	Aflatoxin M_1_ (AFM_1_)	0.00–0.038 *μ*g/L (mean: 0.0068–0.0069 *μ*g/L); wet season: 0.00–0.021 *μ*g/L; dry season: 0.00–0.038 *μ*g/L	Enzyme‐linked immunosorbent assay (ELISA) using Helica AFM_1_ kit (LOD: 5 pg/mL)	[31]
8	240 raw cow milk samples (120 samples collected during wet season and 120 during dry season)	Hulla and Dale districts, Sidama Zone, Southern Ethiopia	Aflatoxin M_1_ (AFM_1_)	0.00–0.038 *μ*g/L (mean: 0.0068 *μ*g/L); wet season: 0.00–0.021 *μ*g/L; dry season: 0.00–0.038 *μ*g/L	Enzyme‐linked immunosorbent assay (ELISA) using Helica AFM_1_ milk kit (LOD: 5 pg/mL)	[32]
9	160 composite samples: Raw milk (*n* = 64), pasteurized milk (*n* = 64), and cottage cheese (*n* = 32) collected from producers, collectors, processors, and retailers	Three regions of Ethiopia: Oromia, Southern Nations, Nationalities, and Peoples (SNNP/Sidama), and Amhara regions (including Wolmera, Bishoftu/Debre Zeit, Asela, Selale; Hawassa, Yirgalem, Dilla, Wolayta; Debre Birhan, Debre Markos, Bahir Dar, Gondar)	Aflatoxin M_1_ (AFM_1_)	Raw milk: 0.003–2.177 *μ*g/L Pasteurized milk: 0.011–1.798 *μ*g/L Cottage cheese: 0.014–0.539 *μ*g/L Overall dairy products: 0.003–2.177 *μ*g/L (mean 0.285 *μ*g/L)	Enzyme‐linked immunosorbent assay (ELISA) using Helica AFM_1_ Assay kit (competitive ELISA); LOD = 0.002 *μ*g/L	[16]

Subsequent studies conducted in different dairy‐producing areas confirmed the widespread occurrence of AFM_1_ in Ethiopian milk. Tadesse et al. [18] reported AFM_1_ concentrations ranging from 0.029 to 2.159 *μ*g/L in 108 raw and pasteurized milk samples marketed in Bishoftu Town. The relatively high mean concentration (0.835 *μ*g/L) suggests a serious contamination problem in intensively managed dairy systems where commercial concentrates may contribute significantly to aflatoxin exposure. Similarly, Kebede et al. [17] detected AFM_1_ in 114 milk samples from the Addis Ababa milk shed, with concentrations ranging from 0 to 0.893 *μ*g/L. Although the overall mean concentration was lower (0.0465 *μ*g/L), some samples exceeded the European Union maximum limit (0.05 *μ*g/L), indicating potential health risks for regular consumers.

Studies from other regions of Ethiopia generally reported lower AFM_1_ levels compared with the Addis Ababa milk shed. In Nekemte City, East Wollega Zone, Nasir and Gemede [29] found AFM_1_ concentrations of 0.01–0.35 *μ*g/L in raw cow milk samples. Similarly, Mesfin et al. [30] reported AFM_1_ levels between 0 and 0.146 *μ*g/L in milk collected from Holeta, Bishoftu, and Hawassa. These regional differences may be associated with variations in feed quality, dairy management practices, agroecological conditions, availability of commercial feed, and storage conditions of feed ingredients. Seasonal variation was investigated by Tefera et al. [31] and Tadele et al. [32] in Sidama Zone, Southern Ethiopia. Analysis of 240 raw cow milk samples collected during wet and dry seasons showed relatively low AFM_1_ contamination (0.00–0.038 *μ*g/L, mean approximately 0.0068 *μ*g/L). However, contamination was slightly higher during the dry season (maximum 0.038 *μ*g/L) compared with the wet season (maximum 0.021 *μ*g/L). This seasonal difference may be explained by increased dependence on stored feeds and crop residues during dry periods, which are more susceptible to fungal growth and aflatoxin accumulation.

A nationwide multiregion study by Zebib et al. [16] provided one of the most representative datasets on AFM_1_ contamination in Ethiopia. The study analyzed 160 composite samples including raw milk (*n* = 64), pasteurized milk (*n* = 64), and cottage cheese (*n* = 32) collected from value chain actors in Oromia, SNNP/Sidama, and Amhara regions. AFM_1_ concentrations ranged from 0.003 to 2.177 *μ*g/L in raw milk, 0.011 to 1.798 *μ*g/L in pasteurized milk, and 0.014 to 0.539 *μ*g/L in cottage cheese. The overall mean contamination level was 0.285 *μ*g/L. Importantly, the study showed that processing did not eliminate AFM_1_ because pasteurized milk retained contamination levels comparable to raw milk. This confirms that AFM_1_ is relatively heat‐stable and cannot be effectively controlled through conventional pasteurization.

Generally, aflatoxin contamination in Ethiopia occurs throughout the food supply chain, driven by preharvest factors such as drought, high temperatures, insect damage, and poor agronomic practices, as well as delayed harvesting, inadequate drying, and improper storage. Poor ventilation, high moisture, mechanical damage, and inadequate sorting further promote fungal growth and toxin production during postharvest handling, transportation, processing, and marketing. Studies have reported increased aflatoxin levels in maize and red pepper during storage and marketing ([14, 22]). In the dairy chain, contaminated animal feed, particularly maize and oilseed cakes, introduces AFB_1_, which is transferred into milk as AFM_1_ and may persist after pasteurization [9, 16]. Therefore, controlling aflatoxin contamination requires an integrated approach involving good agricultural practices, timely harvesting, effective drying, improved storage, hygienic handling and processing, routine food testing, and regular monitoring of animal feed throughout the supply chain.

### 3.5. Aflatoxin Contamination, Regulatory Limits, and Public Health Risks in Maize, Groundnut, Red Pepper, and Milk

The reviewed studies reveal that aflatoxin contamination is a critical food safety challenge in Ethiopia, with levels in major commodities frequently exceeding international regulatory limits (Figure 2). Groundnut showed the highest contamination, with AFB_1_ reaching 1990.86 *μ*g/kg and total aflatoxins up to 11,900 *μ*g/kg, followed by maize (AFB_1_ up to 381.6 *μ*g/kg), red pepper (total aflatoxins up to 98.99 *μ*g/kg), and milk (AFM_1_ up to 4.98 *μ*g/L). These values exceeded established limits, including EU limits of 2 *μ*g/kg for AFB_1_, 4 *μ*g/kg for total aflatoxins, and 0.05 *μ*g/L for AFM_1_ in milk. Major contamination factors included poor drying practices, improper storage, insect damage, contaminated animal feed, and limited monitoring systems. Excessive aflatoxin exposure poses serious risks to human and animal health, including liver cancer, impaired child growth, immune suppression, and reduced livestock productivity. Therefore, integrated aflatoxin management involving improved postharvest handling, feed quality control, routine surveillance, laboratory strengthening, resistant crop varieties, and effective regulatory enforcement is essential to reduce exposure and protect food safety in Ethiopia.

**Figure 2:**
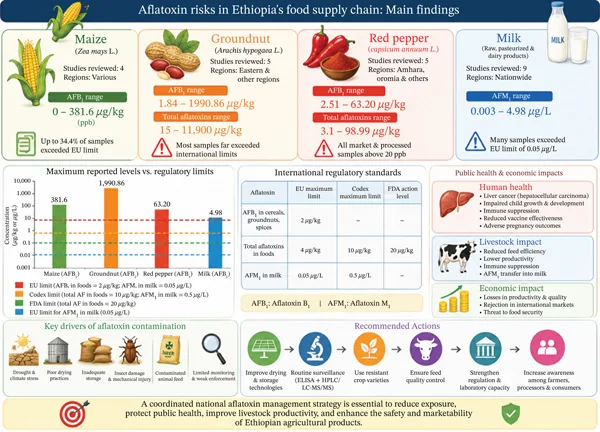
Summary of aflatoxin contamination, regulatory limits, and public health risks in major food commodities of Ethiopia.

## 4. Conclusion

Aflatoxin contamination remains a major food safety challenge in Ethiopia, with maize, groundnut, red pepper, and milk frequently exceeding international regulatory limits. Groundnut showed the highest contamination, followed by maize and red pepper, whereas milk represents an important indirect exposure route through contaminated animal feed. Contamination is primarily associated with poor postharvest handling, inadequate storage, and weak monitoring systems. Reducing aflatoxin exposure requires integrated interventions, including improved drying and storage technologies, routine surveillance using validated analytical methods, resistant crop varieties, feed quality control, strengthened regulatory enforcement, and increased stakeholder awareness. A coordinated national aflatoxin management strategy is essential to protect public health, improve livestock productivity, and enhance the safety and marketability of Ethiopian agricultural products.

### 4.1. Recommendation

Based on the findings of this systematic review, Ethiopia should strengthen aflatoxin prevention and control across the food and feed supply chains through improved pre‐ and postharvest management practices, including timely harvesting, proper drying, sorting, and appropriate storage technologies. The development and adoption of aflatoxin‐resistant crop varieties, routine surveillance of maize, groundnut, red pepper, and milk using validated analytical methods, and improved quality control of animal feed should be prioritized. Furthermore, strengthening food safety regulations, laboratory capacity, regulatory enforcement, and stakeholder awareness through targeted training for farmers, traders, processors, and livestock producers is essential. Coordinated efforts among agricultural, public health, research, and regulatory institutions are also recommended to establish and implement an integrated national aflatoxin management strategy to reduce human and animal exposure and improve food safety in Ethiopia.

## Author Contributions

All authors contributed equally to the conception, literature review, analysis, writing, and revision of this manuscript.

## Funding

No funding was received for this manuscript.

## Disclosure

All authors read and approved the final version of the manuscript prior to submission.

## Ethics Statement

The authors have nothing to report.

## Consent

The authors have nothing to report.

## Conflicts of Interest

The authors declare no conflicts of interest.

## Data Availability

The data that support the findings of this study are available from the corresponding author upon reasonable request.
